# The Value of the Nurse Navigator in Complex Cancer Care: A Scoping Review

**DOI:** 10.3390/healthcare13131585

**Published:** 2025-07-02

**Authors:** Kaitlin Muzio, Jenna Hiemstra, Maya Morton-Ninomiya, Dana Toameh, Emma Nicholson, Kathryn V. Isaac

**Affiliations:** 1 School of Public Health Sciences, University of Waterloo, Waterloo, ON N2L 3G1, Canada; 2 Faculty of Medicine and Health Sciences, McGill University, Montreal, QC H3A 0G4, Canada; 3 Division of Plastic Surgery, Department of Surgery, University of British Columbia, Vancouver, BC V6T 1Z4, Canada

**Keywords:** nurse navigators, cancer care, patient experience, literature review, oncology nursing, patient-centered care

## Abstract

**Background/Objectives**: Many Canadians experience challenges navigating the healthcare system during their cancer care. Nurse navigators are uniquely positioned to support patients with their clinical expertise in oncology and patient care, but they have not been widely implemented. This study aimed to examine the impact of nurse navigators and barriers to successful implementation of a nurse navigator program. **Methods**: MEDLINE, EMBASE, and Web of Science databases were searched for articles examining the role of nurse navigators in cancer care. The data was extracted on study design, patient characteristics, nurse navigators’ responsibilities, outcomes, barriers to success, and recommendations for implementing nurse navigator programs. Content analysis was used to identify common themes. **Results**: Of 1787 articles identified, 44 articles met the inclusion criteria and underwent data extraction. Nurse navigator responsibilities included patient education, psychosocial support, clinical assessment, care coordination, patient advocacy, and improving workflows. Most studies reported significant benefits from nurse navigator programs, including patient-centered care, satisfaction with the healthcare system, reduced patient distress, healthcare provider support, and enhanced patient monitoring. Barriers included a lack of understanding of the role, overwhelmed nurse navigators, and inefficient healthcare system workflows. Recommendations for future nurse navigator programs include providing personalized support to patients, encouraging integrated healthcare teams, and permanent funding. **Conclusions**: Nurse navigator programs improve cancer patients’ experiences and the efficiency of cancer care delivery. Implementation necessitates integration into the healthcare team and longitudinal financial and professional support of nurse navigators.

## 1. Introduction

Cancer is one of the leading causes of death in Canada and the health burden from this disease is expected to worsen over the century [1,2]. In their quests to deliver high-quality cancer care, healthcare systems across Canada are increasingly overwhelmed, resulting in long wait times and stress amongst healthcare providers, indicating a need for greater healthcare system support [3,4]. Patients may be tasked with learning about their cancer diagnosis and treatment options, consulting with numerous healthcare specialists, managing symptoms, and making life-changing decisions, without consistent guidance and coordination of their care [5,6,7]. Patients often report confusion and isolation as they attempt to self-navigate through this fragmented healthcare system [7,8]. Nurse navigation programs emerged in the 1990s to address these challenges and provide patients with guidance and emotional support throughout their cancer care [7,9,10]. These programs utilize nurses’ expertise in cancer care and knowledge of the healthcare system, with the trained nurse navigator advocating for and supporting patients while coordinating patients’ care, by communicating with the healthcare team and reducing barriers [11]. Nurse navigator programs are diverse in their offerings, with some spanning the entire cancer care journey while others are only offered at specific time points (i.e., during chemotherapy) [12,13]. Additionally, some nurse navigator programs are restricted to specific patient populations, such as specific cancer types or high-risk individuals [14].

Literature reviews to date have focused on the importance of patient navigation in healthcare, finding significant benefits for patients, providers, and healthcare systems when implemented in oncologic care settings [15,16,17].

However, there is a notable gap in our understanding of how to best integrate nurse navigators in public healthcare systems, given that previous reviews have primarily focused on private settings, predominantly in the American healthcare system [18,19]. Reviews were also published prior to 2020, suggesting that there is new evidence following the COVID pandemic, which may provide important insights for contemporary considerations.

The aim of this scoping review was to investigate if and how nurse navigator programs improve cancer care delivery in public healthcare systems, and what barriers prevent the successful implementation of nurse navigator programs. Furthermore, we aimed to provide recommendations for developing and implementing effective nurse navigator programs in the setting of complex cancer care delivery in public healthcare systems. This scoping review will address the gaps in implementing nurse navigators in public healthcare systems and examine the current literature that has yet to be reviewed.

## 2. Materials and Methods

This scoping literature review investigated nurse navigator programs’ effects on complex cancer care and how nurse navigator programs were successfully implemented in public healthcare systems. Complex cancer care is defined in this scoping review as cancer care that necessitates multimodal treatment and corresponding specialists (i.e., radiation, chemotherapy and surgery) as compared to simple skin cancer that may just require surgery. A protocol for the scoping review was published on the Open Science Framework on 10 June 2022, and can be found at: https://doi.org/10.17605/OSF.IO/CNJ24. This scoping review is compliant with the PRISMA guidelines for scoping reviews.

A systematic search was conducted in November 2024 in the MEDLINE, EMBASE, and Web of Science databases. Searches were limited to studies originally published in English in academic journals but were not limited to specific time frames or sample sizes. The search was focused on countries with public healthcare systems. Additional studies were identified by manually searching the reference lists of included articles. Gray literature sources were queried with relevant keywords. Search strategies used for each database are detailed in Table A1, with each search string using keywords such as “patient navigation”, “malignancy”, “oncology”, and “public healthcare”. To account for the fact that the nurse navigator role is not well-defined, the search included nurses with different role titles that may qualify as nurse navigators, such as pivot nurses and nurse care coordinators.

In the first stage of screening, search results were uploaded to Covidence systematic review software (Veritas Health Innovation, Melbourne, VIC, Australia, available at www.covidence.org) to facilitate the selection of articles and remove duplicates. Reviewers independently screened the titles and abstracts of articles against the predetermined inclusion and exclusion criteria (Table A2). Studies were required to report on the role of nurse navigators, or nurses operating within the scope of patient navigation, in cancer care involving multiple disciplines. Studies were required to take place in Canada or another publicly funded healthcare systems. Studies that focused on pediatric patients, patients without cancer diagnoses, or administration in private healthcare systems were excluded. Ineligible study designs included reviews, editorials, economic evaluations, and models of planned nurse navigator programs. In the second stage, reviewers read the full text of the remaining articles and reviewed them against the predetermined inclusion and exclusion criteria. In both stages, conflicts were discussed, and any disagreements were resolved by a third reviewer. All agreed-upon articles were included in the review.

All studies selected for inclusion underwent data extraction by one coder, with conflicts and consensus addressed by one additional coder. The coders extracted data on study design, patient characteristics, characteristics of the nurse navigator program, patient and healthcare system outcomes, barriers to program success, and recommendations for implementation of nurse navigator programs. Following data collection, content analysis was used to identify common themes. Extracted data was divided into meaning units, which were then sorted into key themes addressing successful attributes of nurse navigator programs, challenges these programs face and recommendations for implementation [20].

## 3. Results

### 3.1. Sources of Evidence

**Figure 1:**
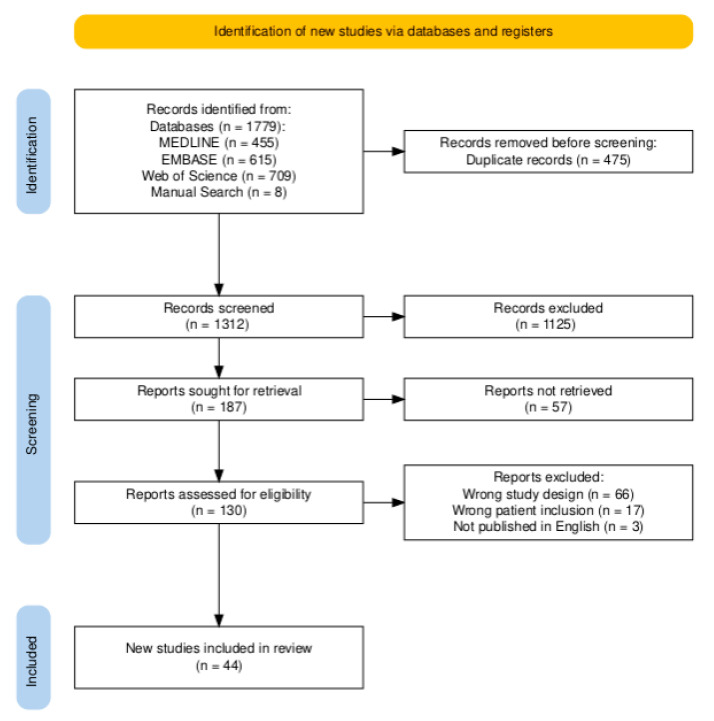
PRISMA diagram of articles included in scoping review, developed with PRISMA Flow Diagram Tool [21]. Of 1312 unique articles identified, 44 studies were included in the scoping review.

The search returned 1779 articles related to nurse navigators in cancer care, and 8 identified through manual search, shown in the PRISMA diagram in Figure 1 [21]. Once duplicates were removed (*n* = 475), 1312 articles underwent title and abstract screening and 130 articles proceeded to full-text review. A total of 44 articles met inclusion criteria and underwent data extraction and thematic coding for this review. No articles were identified for inclusion from the gray literature search.

### 3.2. Study Characteristics

The characteristics of the 44 articles included in this review are summarized in Table 1. Most articles probed patient and provider perceptions of nurse navigator programs through interviews, focus groups, chart reviews, or surveys. Other data collection methods included researcher observations during site visits, workload measures, and consultations with nurse navigators and clinical experts. Some nurse navigator programs were offered to patients with specific types of cancer (n = 26, 59%), while the remainder of articles were non-specific. A majority of articles (n = 30, 68%) were focused on nurse navigator programs implemented in Canada.

### 3.3. Characteristics of Nurse Navigator Programs

**Figure 2:**
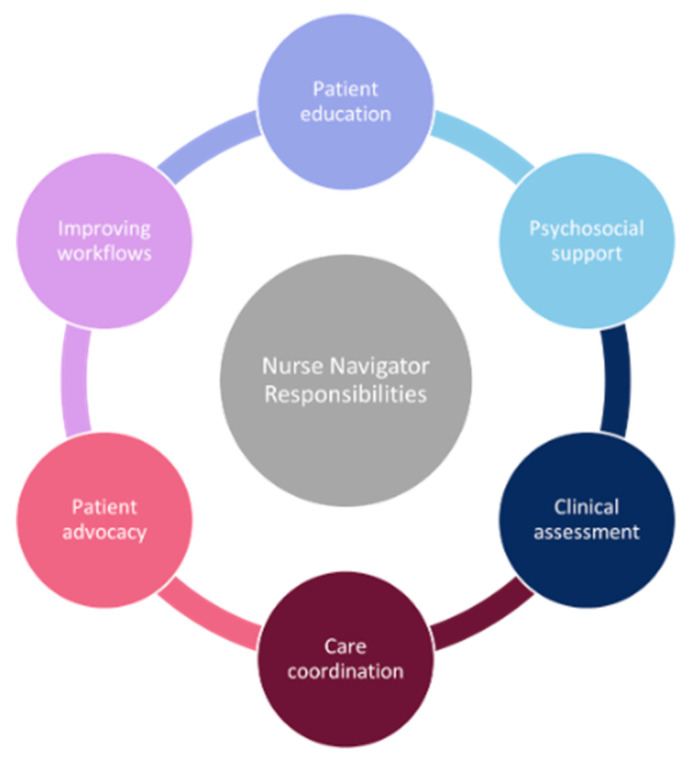
Key responsibilities of nurse navigators include patient education, psychosocial support, clinical assessment, care coordination, patient advocacy and improving workflows.

Across the articles reviewed, six common responsibilities of nurse navigators emerged (Figure 2). Patient education involved the nurse navigator providing additional information to patients regarding their disease, symptom management, and treatment, and supporting shared decision-making. Nurse navigators provided psychosocial support to patients, where they would actively listen, counsel, and be available to support based on patient needs. Clinical assessment involved performing symptom screening, patient monitoring, and escalating health concerns to other healthcare professionals. Care coordination referred to communication with other healthcare team members, referrals of patients to additional support, and addressing patient-identified barriers to treatment. Nurse navigators’ patient advocacy was evident when they attended patients’ medical appointments and emergency room visits to ensure patients’ wishes were respected. Nurse navigators improved workflows within the healthcare team by training staff and creating innovative solutions to administrative inefficiencies within the healthcare system. None of the nurse navigator programs reviewed included all six responsibilities, but most included two or more in any combination.

Additionally, several studies described nurse navigator responsibilities that did not span the full episode of care. Some articles described nurse navigator programs available to patients preoperatively, wherein nurse navigators coordinated care and diagnostic activities from referral to their first appointment [22,23,24]. Other studies described nurse navigator programs that were offered to patients only for the duration of their chemotherapy treatment [25,26,27]. Some nurse navigator programs had narrowed administrative scopes, focusing on coordinating patient referrals to a cancer center [28], or offered by private health insurance plans to help coordinate care in a public healthcare system [29].

### 3.4. Successful Components of Nurse Navigator Programs

Most articles reported benefits from implementing nurse navigator programs, both for patients and the healthcare system. Analysis of the reported positive impacts revealed five distinct themes related to successful aspects of nurse navigator programs (Table 2). Only 1 study (2.5%) reported no significant benefit from the nurse navigator program [30]. Importantly, in this study, the benefits were assessed by comparing nurse navigators who received training in cancer symptom management but did not have experience working in oncology care settings, to the standard of care which involved oncology nurses with extensive clinical knowledge [30].

### 3.5. Barriers to Implementing Nurse Navigator Programs

Although the nurse navigator programs showed significant benefits, their implementation and sustainability faced notable challenges (Table 3).

Some studies reported limited participation in nurse navigator programs, attributed to physicians not understanding the scope of the nurse navigator’s role which limited limiting referrals to the program [12,28,29,31,32,33]. Health professionals also asked nurse navigators to do work outside their defined role, or were reluctant to engage with the nurse navigator [34,35]. Inappropriate referral of patients was characterized by too many patients referred, surpassing program capacity, or patients who should have been assisted by a nurse navigator were missed [36]. Within the overwhelmed nurse navigator theme, some studies highlighted issues with a lack of sufficient funding to establish the nurse navigator program [24,33]. Additionally, nurse navigators reported a lack of adequate resources such as access to electronic charts, offices to meet with patients, or assessment tools, restricting their ability to carry out their responsibilities [7,34,37].

The misaligned patient expectations barrier was identified in studies where nurse navigators did not initiate contact with patients or they were only present at specific points in the patient care journey [32,38]. Patients also confused the nurse navigator for a student doctor or were worried about burdening the nurse navigator, and therefore did not utilize the nurse navigators [27,39,40]. Gaps in communication and integration between healthcare teams made following up with patients challenging, especially when patients’ care was spread across several departments or health authorities [31,32,41].

### 3.6. Recommendations for Implementing Nurse Navigator Programs

Based on the successes and barriers identified through nurse navigator program assessment, proposed recommendations are summarized for implementing future nurse navigator programs or refining existing ones (Table 4). These recommendations apply to nurse navigator programs implemented within public healthcare systems.

Nurse navigator programs were best received by patients when they offered flexible delivery of care (i.e., in-person, telephone, virtual), and when the nurse navigator initiated contact with the patient [26,38,42,43,44]. Articles highlighted the importance of nurse navigators assessing all areas of patients’ needs and adapting the delivery of information based on the patient’s understanding [32,45,46]. Studies also emphasized the nurse navigator offering emotional support, empowerment, and compassion to facilitate patient-centered care [25,37,47,48]. For nurse navigators to effectively support both the patient and clinical team, they require access to appropriate training, formal assessment tools, and electronic medical records [26,34,35,49,50]. They also require sufficient staff and funding to ensure the nurse navigator program meets its demands and is sustainable [22,50,51,52]. Integrated healthcare teams that encourage communication and collaboration between providers, healthcare teams, and allied health professionals facilitate the success of nurse navigator programs [31,32,37,51]. Clearly defining the nurse navigator role by educating healthcare teams and patients on the role of nurse navigators will maximize the effective usage of the program [33,34,40,48]. Programs should also create clear and consistent referral pathways so that the target group of patients is reached [9,31,49,51].

## 4. Discussion

This review supports the implementation of nurse navigators for the provision of high-quality patient-centered oncologic care in public health systems. Nurse navigators enhance care through the delivery of patient education, psychosocial support, clinical assessment, care coordination, patient advocacy, and improving workflows. Many included studies examined nurse navigator programs in the Canadian healthcare system, as it is an active area of evaluation in a public healthcare system. We identified that a limited understanding of the nurse navigator role led to patient confusion and misuse of the nurse navigators, restricting the benefits of the program. Researchers have attempted to address the lack of role clarity by creating frameworks for nurse navigators, proposing rigorous criteria centered around facilitating continuity of care and promoting patient empowerment [7], and creating orientation modules to train new nurse navigators [33]. Explicitly defining the role of nurse navigators and providing systematic training in navigation strengthens nurse navigators’ ability to support patients and function within an interdisciplinary team [17].

When appropriately integrated, nurse navigators positively impact patient care; reducing patient distress, improving symptom management, and decreasing complications [53,54], translating to a measurable improvement in quality of life [55,56]. It is well documented that patient satisfaction with care is linked to self-reported physical well-being, with minimal complications and symptom burden associated with better patient experiences [57,58]. Patients with access to nurse navigators had significant improvements in psychological well-being when assessed with standardized measures such as HADS (Hospital Anxiety and Depression Scale), RSCL (Rotterdam Symptom Checklist), Symptom Distress Scale [30,41,42,59], although few studies used quantitative outcome measurements. It should be noted that the ability of nurse navigator programs to impact patient distress and health outcomes will depend on the scope of the role, programs with minimal patient interaction or short duration may not result in the same outcomes.

This review also highlighted the benefits that nurse navigation programs can bring to clinicians when appropriately implemented, reducing administrative burdens and fostering communication between interdisciplinary team members [60,61,62]. Nurse navigators also benefited healthcare systems, in one study mediating a decrease in average time from diagnosis to surgery from 47 days to 41 days [28]. Evidence from a cost-utility analysis provided support for improved healthcare system functioning, demonstrating that use of nurse navigators was a cost-effective intervention in the long term despite initial program set-up costs [63]. In the current healthcare climate, which is marked by discussions of rising healthcare costs and burnt-out clinicians, nurse navigators could play an important role [64]. While it may seem like an added financial cost, the long-term impact favors nurse navigator implementation to decrease clinician burden, increase time for providers to focus on patient care, and provide cost savings for the healthcare system.

This scoping review has identified a need for future research to investigate novel ways to address the barriers to implementing nurse navigators in public healthcare systems. Given the benefits that nurse navigators may provide in improving healthcare system functioning, health economic evaluations should be used to confirm whether nurse navigators provide net cost savings to the healthcare system. Prospective studies are needed to address the paucity of quantitative evidence on the impact of nurse navigator programs, and could include measurable outcomes such as psychological well-being (using standardized measures such as HADS or RSCL) or healthcare system process outcome measures (i.e., time to surgery). There is also a gap in information on the satisfaction of providers with nurse navigator programs, an area that warrants further investigation.

Despite the strengths of our rigorous study methodology, there are limitations inherent to literature reviews. The limited critical appraisal of evidence hinders our ability to comment on the effectiveness of the interventions. With our focus on publicly funded healthcare systems, this study’s results may not be generalizable to privately funded nurse programs. Since most of the studies included in this review were from Canada, this may influence the generalizability of the results. Additionally, the exclusion of pediatric cancer patients and privately funded healthcare systems means that the results from this review may not be applicable to nurse navigator programs for these populations. The synthesis of our findings is limited to the review of evidence in published studies. There is limited evidence to report on the impact of nurse navigator programs on provider satisfaction, clinical outcomes, and/or healthcare costs. As well, there was a lack of information on facilitators who support the implementation of nurse navigator programs, making it difficult to explain how nurse navigator programs are started. Moreover, the nurse navigator role, or parts of the role, may be taken upon by physicians or other allied health professionals. Thus, the utility and impact of the nurse navigator, as reported in this review, must be considered in each local setting.

## 5. Conclusions

This study supports that nurse navigator programs can positively impact both patients and the healthcare system when appropriately integrated. However, when there is a lack of funding or understanding of the role, it can inhibit the program’s success. Healthcare institutions aspiring to implement nurse navigator programs are encouraged to develop clear definitions of the role, establish consistent support for the nurse navigator, and integrate nurse navigators within healthcare teams.

## Figures and Tables

**Table 1:** General characteristics of included articles (n = 44).

General Characteristics	Sub-Categories	Number (*n* = 44)	Percentage
Study Design	Cross-Sectional	25	57
	Interventional	12	27
	Retrospective	6	14
	Mixed Methods	1	2
Focus of Study	Assessing existing program	27	61
	Implementing new program	16	36
	Refining existing program	1	2
Data Collection Method	Interview	23	52
	Survey	20	45
	Chart review	9	20
	Focus group	4	9
	Other	5	11
Country of Publication	Canada	30	68
	Australia	6	13
	United Kingdom	2	5
	France	2	5
	Germany	2	2
	Sweden	1	2
	Denmark	1	2
Specific Cancer(s) Studied	Breast	20	45
	Lung	5	11
	Colorectal	4	9
	Prostate	2	5
	Brain	1	2
	Esophageal	1	2
	Liver	1	2
	Ovarian	1	2

**Table 2:** Benefits observed in some nurse navigator programs.

Theme	Description	Frequency (*n*)
Patient-centered care & patient empowerment	Patients appreciate nurse navigators’ predictability, humanization and coherence Improved patient comprehension, shared decision-making, and medication adherence Patients more willing to share intimate details of lives	32
Patient satisfaction with healthcare	Decreased wait-times Improved coordination of care and access to supportive services Fewer complications (i.e., ER visits, re-admission)	30
Reduced patient distress	Reduced anxiety, depression, worry, stress Improved use of coping strategies, problem-solving to adjust to illness	24
Healthcare provider support	Decreased workload and burden on other providers Encouraged effective communication and cooperation between providers Improved relationships and information sharing between patients and providers Improved clinical workflows	18
Patient monitoring	Enhanced patient monitoring Improved symptom management and quality of life Fewer unwanted side effects	18

**Table 3:** Barriers to nurse navigator implementation.

Theme	Description	Frequency (*n*)
Lack of understanding of role	Misuse of nurse navigators Challenges defining the nurse navigator’s role	11
Inappropriate referral to the nurse navigator program	Over-referral of patients without need of additional support Not all patients who would benefit are referred	9
Overwhelmed nurse navigators	Demand for program outweighs capacity Insufficient resources and funding Concerns for nurse navigator stress and burnout	8
Misaligned patient expectations from nurse navigators	Patient confusion over role of nurse navigators in their care Patients wanted additional contact, follow-up, and information from nurse navigator	7
Inefficient healthcare system workflows	Inadequate communication between healthcare providers Difficulty accessing patients’ medical information	6

**Table 4:** Recommendations for successful nurse navigation programs.

Recommendations for Successful Nurse Navigation Programs
Provide personalized patient support and proactively contact patients Create trusting relationships with patients based in compassion and empathy Establish permanent funding and adequate support for nurse navigators Encourage integrated healthcare teams and communication between providers Clearly define the nurse navigator program and the roles of the nurse navigators

## Data Availability

No new data were created or analyzed in this study. Data sharing is not applicable to this article.

## Appendix A

**Table A1:** Search Strategies of MEDLINE, EMBASE, and Web of Science.

**MEDLINE (Ovid)** exp Breast Neoplasms/ ((breast* or mammar*) adj2 (neoplasm* or cancer* or oncolog* or carcinoma* or tumo#r* or malignan*)).tw,kf. 1 or 2 exp Neoplasms/ (oncolog* or cancer* or carcinoma* or tumor* or tumour* or neoplasm* or metasta* or malignan*).tw,kf. 4 or 5 exp Oncology Nursing/ or exp Patient Navigation/ (nurs* navigator* or patient* navigat* or pivot nurs* or Infirmiere Pivot en Oncologie or nurs* care coordinat*).tw,kf. (nurs* adj3 (navigat* or care coordinat* or counsell* or guid*)).tw,kf. 7 or 8 or 9 exp Canada/ (Canad* or “British Columbia” or “Colombie Britannique” or Alberta* or Saskatchewan or Manitoba* or Ontario or Quebec or “Nouveau Brunswick” or “New Brunswick” or “Nova Scotia” or “Nouvelle Ecosse” or “Prince Edward Island” or Newfoundland or Labrador or Nunavut or NWT or “Northwest Territories” or Yukon or Nunavik or Inuvialuit).tw,kf. 11 or 12 (public* adj4 healthcare).tw,kf. exp United Kingdom/ exp Australia/ exp “Scandinavian and Nordic Countries”/ exp France/ exp Germany/ 14 or 15 or 16 or 17 or 18 or 19 3 and 10 and 13 6 and 10 and 13 3 and 10 and 20
**EMBASE (Ovid)** exp Canada/ (Canad* or “British Columbia” or “Colombie Britannique” or Alberta* or Saskatchewan or Manitoba* or Ontario or Quebec or “Nouveau Brunswick” or “New Brunswick” or “Nova Scotia” or “Nouvelle Ecosse” or “Prince Edward Island” or Newfoundland or Labrador or Nunavut or NWT or “Northwest Territories” or Yukon or Nunavik or Inuvialuit).tw,kf. 1 or 2 (public* adj4 healthcare).tw,kf. exp United Kingdom/ exp Australia/ exp Germany/ exp France/ exp Scandinavia/ 4 or 5 or 6 or 7 or 8 or 9 (nurs* navigator* or patient* navigat* or pivot nurs* or Infirmiere Pivot en Oncologie or nurs* care coordinat* or oncolog* nurs*).tw,kf. (nurs* adj3 (navigat* or care coordinat* or counsell* or guid*)).tw,kf. (patient* adj3 (navigat* or care coordinat*)).tw,kf. 11 or 12 or 13 exp breast cancer/ ((breast* or mammar*) adj2 (neoplasm* or cancer* or oncolog* or carcinoma* or tumo#r* or malignan*)).tw,kf. 15 or 16 malignant neoplasm/ or neoplasm/ (oncolog* or cancer* or carcinoma* or tumor* or tumour* or neoplasm* or metasta* or malignan*).tw,kf. 18 or 19 3 and 14 and 17 3 and 14 and 20 10 and 14 and 17
**Web of Science Core Collection** (((((ALL=(patient navigation)) OR ALL=(nurse navigator)) OR ALL=(pivot nurse)) OR ALL=(patient navigator)) OR ALL=(nurse care coordinator)) OR ALL=(Infirmiere Pivot en Oncologie) (((((ALL=(breast cancer)) OR ALL=(breast neoplasm)) OR ALL=(breast oncology)) OR ALL=(breast carcinoma)) OR ALL=(breast malignancy)) OR ALL=(breast tumor) #2 AND #1 and CANADA (Countries/Regions) (((((ALL=(cancer)) OR ALL=(neoplasm)) OR ALL=(oncology)) OR ALL=(carcinoma)) OR ALL=(malignancy)) OR ALL=(tumor) #4 AND #1 and CANADA (Countries/Regions) #2 AND #1 and GERMANY or AUSTRALIA or ENGLAND or FRANCE or SWEDEN or DENMARK or IRELAND or WALES or FINLAND or ICELAND (Countries/Regions)

## Appendix B

**Table A2:** Inclusion and exclusion criteria.

Inclusion Criteria	Exclusion Criteria
Studies specifically reporting on patients who have been diagnosed with breast cancer, or another type of cancer that necessitates multidisciplinary care, including chemotherapy, radiation, and surgery as treatment options. Studies that report on the role of nurse navigators in cancer care. Studies must include adult patients (18+ years). Studies must have country of origin with publicly funded healthcare system Published in the English language. No time limits.	Studies reporting patients without a cancer diagnosis (i.e., Patients with other chronic disease). Studies reporting non-adult patients (under 18 years). Studies focusing on nurse navigator programs in privately funded healthcare systems. Studies reporting on alternative navigation programs (i.e., Peer navigators). Studies that are systematic reviews or meta-analyses. Not published in the English language. Study protocols or abstracts.
